# A Study to Evaluate the Role of Three-Dimensional Pseudo-Continuous Arterial Spin Labelling in Acute Ischemic Stroke

**DOI:** 10.7759/cureus.44030

**Published:** 2023-08-24

**Authors:** Smitha Ravula, Chandrashekar Patil, Prashanth Kumar KS, Raja Kollu, Abdul Raheem Shaik, Rohit Bandari, Rajesh Songa, Vasudha Battula, Samuel Paul Dhinakar Arelly, Ragini Gopagoni

**Affiliations:** 1 Radiodiagnosis, Malla Reddy Medical College for Women, Hyderabad, IND; 2 Radiology, New Medical Centre (NMC) Speciality Hospital, Abu Dhabi, ARE; 3 Neurology, Malla Reddy Narayana Multispeciality Hospital, Hyderabad, IND; 4 Pediatrics, Ankura Hospital Hyderabad, Hyderabad, IND; 5 General Surgery, Christian Medical College, Vellore, IND; 6 Internal Medicine, Malla Reddy Institute of Medical Sciences, Hyderabad, IND

**Keywords:** diffusion weighted imaging ( dwi ), penumbra, window period, diffusion perfusion mismatch, arterial spin labeling

## Abstract

Introduction

Magnetic resonance imaging (MRI) is well known to detect ischemic brain tissue and evaluate the tissue vulnerable to infarction. Diffusion-weighted imaging (DWI) has been a mainstay of stroke evaluation but has a few shortcomings, as it generally indicates only the core of ischemia and does not provide information regarding the tissue at risk or the ischemic penumbra surrounding the infarct. Perfusion imaging identifies brain tissue that has reduced blood flow as a potential target for reperfusion therapy. Arterial spin labelling (ASL) is a new non-invasive, non-contrast MRI perfusion sequence used to detect areas of hypoperfusion qualitatively and quantitatively and also identify the area at risk, i.e., the penumbra, in acute ischemic stroke. The most important component of the imaging is to determine the ischemic penumbra. One of the working definitions of penumbra is brain tissue that is ischemic but not yet infarcted and is at risk of further damage unless the flow is rapidly restored. Hence, perfusion-diffusion mismatch provides a realistic target for potential intervention. The aim of our study is to assess the role of ASL imaging in identifying the penumbra and providing insight into the management of acute ischemic stroke.

Materials and methods

Patients who presented with symptoms of acute ischemic stroke were included in the study, and an MRI stroke protocol comprising DWI, fluid-attenuated inversion recovery (FLAIR), ASL, and magnetic resonance* *angiogram (MRA) sequences was done. Post-thrombolysis, a follow-up MRI was done using DWI, ASL, and MRA to see the restoration of perfusion in the ischemic penumbra. Three-dimensional pseudo-continuous ASL (in our study, ASL refers to pseudo-continuous ASL) is included in the stroke protocol in cases of acute ischemic stroke and assessed qualitatively.

Results

Our study included 43 patients (n = 43), of whom 39.5% (17 patients) belong to the age group of 51-60 years and 2.3% (one patient) are in the age group of 21-30 years. All 43 cases demonstrated DWI-FLAIR mismatch, suggestive of ischemic stroke within the window period, and all 43 cases showed DWI-ASL mismatch, suggestive of a large yet potentially salvageable peri-infarct ischemic penumbra. The most common territory involved was the middle cerebral artery (MCA), and the posterior cerebral artery (PCA) was the least commonly involved territory. We had one case involving the MCA-PCA watershed zone.

Conclusion

Arterial spin labelling is a novel, non-invasive, non-contrast MRI sequence with the capability to provide qualitative information regarding the salvageable ischemic penumbra, and timely management prevents the progression of the penumbra. The incorporation of ASL as part of the standard neuroimaging protocol aids in the management of acute stroke, giving insight into the prediction of outcome.

## Introduction

Ischemic stroke can be defined as a neurological deficit due to cerebral infarction. Stroke is one of the most devastating neurological conditions and accounts for close to 5.5 million deaths worldwide every year, with 13.6 million incident cases causing a loss of 116.4 million disability-adjusted life years. According to recent statistics in southeast Asian countries, there are 0.9 million deaths and 1.5 million incident cases causing 22.2 million disability-adjusted life years, whereas in India, there are 0.6 million deaths and 1.1 million new cases causing 16.3 million disability-adjusted life years [1].

Acute ischemic stroke occurs within 24 hours of symptom onset. In acute stroke, it is no longer sufficient to simply detect ischemia but also try to evaluate the area at risk, reperfusion or recanalization status, and predict eventual hemorrhagic transformation [2]. The magnetic resonance imaging (MRI) Diffusion-weighted imaging (DWI) sequence is highly sensitive for detecting small and early infarcts that are irreversible. The DWI sequence will not provide information regarding the reversible ischemic penumbra.

The penumbra is a dynamic entity that exists within a narrow range of perfusion pressure, and the duration of the delay in recanalization is inversely related to the size of the penumbra [3]. Penumbra is generally accepted as the volume of the brain showing a larger perfusion defect than a diffusion defect (DWI mismatch). Penumbra is potentially salvageable with reduced tissue perfusion, but not beyond the level of irreversibility. It is the ischemic penumbra that benefits more from thrombolytic intervention if it is identified in a timely fashion. Timely reperfusion prevents the progression of the disease.

Arterial spin labelling (ASL) is an effective MR technique for visualizing perfusion and quantifying cerebral blood flow (CBF). ASL perfusion imaging uses blood as an endogenous contrast agent by magnetically labelling it with radiofrequency pulses and does not require gadolinium-based contrast agents. The perfusion contrast is given by the difference in magnetization induced by the exchange of these labelled spins at the brain tissue level with a non-labelled control image.

Advantages of ASL

It is non-contrast, non-invasive, safe to use in paediatric patients, and can be used in patients with renal dysfunction [4].

In routine clinical practice, perfusion is estimated with dynamic susceptibility contrast (DSC) imaging and dynamic contrast enhancement (DCE T2*). Employment of gadolinium-based contrast agents is, however, limited due to the likelihood of inducing nephrogenic systemic fibrosis in patients with poor renal function [5-6].

Detection of regions of mild or moderate ischemia and ischemic penumbra requires the assessment of CBF, cerebral blood volume (CBV), or mean transit time (MTT), which provide quantitative information about the tissue at risk. The CBF of normal brain parenchyma is >60 ml/100 g of brain tissue/min; the CBF of tissue at risk is >20 ml/100 g of brain tissue/min but not <10 ml/100 g of brain tissue/min; whereas the CBF of the infarct region is < 10 ml/100 g of brain tissue/min [7].

The aim of our study is to assess the use of ASL imaging in detecting the penumbra and offering insight into the management of acute ischemic stroke.

## Materials and methods

Study design

This is a hospital-based observation study done for a period of one and a half years.

Methodology

Our study included all adult patients with acute ischemic stroke who presented within the window period (within four and a half hours of the onset of symptoms) and were referred to the Department of Radiodiagnosis at Malla Reddy Narayana Multispecialty Hospital, a tertiary care hospital attached to Malla Reddy Medical College for women. DWI, fluid-attenuated inversion recovery sequence (FLAIR), 3D pseudo-continuous arterial spin labelling (pCASL), and magnetic resonance angiogram (MRA) are the main sequences used in our study to scan the patients. Post-thrombolysis ASL and MRA were acquired to demonstrate the opening up of the vessel and restoration of perfusion in the ischemic penumbra, and the same data were collected.

Information collected

Data were collected by the investigator through interviews and medical record reviews. Detailed clinical history, demographic information, and cardiovascular risk factors, including age, sex, hypertension, diabetes mellitus, current smoking, and previous history of stroke, were collected.

Inclusion criteria

Patients with acute ischemic stroke who came to the hospital within the window period had a DWI-FLAIR mismatch.

Exclusion criteria 

Patients with subacute, chronic, and lacunar infarcts were excluded from our study.

Image accquisition

The standard clinical stroke protocol was performed using the SignaHDxT GE Healthcare 1.5T, 16-channel head coil MRI scanner system. DWI was performed with a b value of 1500 s/mm^2^, a repetition time (TR) of 10,300 ms, an echo time (TE) of 98 ms, a voxel size of 0.6 × 0.6 × 3.0 mm^3^, a slice thickness of 3.0 mm, and an acquisition time of 46 sec. 

The ASL product sequence included a multi-inversion time (TI) pseudo-continuous ASL (pCASL) with the following details: flow alternating inversion recovery (FAIR) labelling using a Q2TIPS saturation scheme, anterior-posterior labelling, six post-labelling delays (PLD) time points between 600 ms and 3,600 ms, TR: 4,800 ms, TE: 36.52 ms. The image readout was performed using single-shot 3D gradient and spin echo (GRASE) echo planar imaging (EPI) with the following parameters: voxel size of 4.0 × 4.0 × 5.0 mm^3^ and slice thickness of 5.0 mm. These parameters were chosen to allow a clinically relevant acquisition time of 4 minutes. The total stroke protocol acquisition time is about eight to nine minutes.

## Results

This study included 43 patients, 39.5% (17 patients) belonging to the age group of 51-60 years, and 2.3% (one patient) in the age group of 21-30 years (Table 1). Out of them, the majority 69.8.3% (30) are males, and 30.2% (13) are females (Table 2). 45% (27) of cases are hypertensive, 28.3% (17) are diabetic, 26.7% (16) have coronary artery disease, 69.2% are alcoholics, and 30.8% are smokers (Table 3).

**Table 1:** Showing age distribution of the study population.

Age (years)	Number of cases	Percentage
21–30	1	2.3%
31–40	4	9.3%
41–50	7	16.3%
51–60	17	39.5%
61–70	9	20.9%
>70	5	11.6%

**Table 2:** Showing gender distribution in the study population.

Gender	Number	Percentage
Male	30	69.8%
Female	13	30.2%

**Table 3:** Showing comorbidities in the study population.

Comorbidities	Number	Percentage
Hypertension	27	45%
Diabetes mellitus	17	28.3%
Coronary artery disease	16	26.7%
Consumption of alcohol	27	69.2%
Smoking	12	30.8%

Out of the 43 patients, 58.1% (36) presented with complaints of weakness, 38.7% (24) presented with slurring of speech, and about 3.2% (2) presented with other symptoms; one patient presented with excessive sweating and chest pain, and another presented with giddiness (Table 4). 66.7% (24) presented with weakness on the right side, and 33.3% (12) had left-sided weakness.

**Table 4:** Symptoms within the study group.

Symptoms	Number	Percentage
Weakness of upper or lower limbs	36	58.1%
Slurring of speech	24	38.7%
Other symptoms	2	3.2%

All 43 cases showed diffusion restriction on DWI and did not show corresponding hyperintense areas on FLAIR (i.e., DWI-FLAIR mismatch) and showed DWI-ASL mismatch, i.e., diffusion perfusion mismatch (representing peri-infarct ischemic penumbra). In our study, the middle cerebral artery (MCA) was the most commonly involved territory in about 84% (41) of patients; 4% (2) of patients’ posterior cerebral artery (PCA) was involved; 6% (3) of patients' anterior cerebral artery (ACA); 4% (3) of patients MCA-ACA watershed zone; and 2% (1) of patients MCA-PCA watershed zone was involved (Table 5).

**Table 5:** Showing territories involved in the study group. MCA: middle cerebral artery, ACA: anterior cerebral artery, PCA: posterior cerebral artery.

Territories involved	Number	Percentage
MCA	41	84%
ACA	3	6%
PCA	2	4%
MCA-ACA watershed	2	4%
MCA-PCA watershed	1	2%

Patients included in the study underwent an MRI stroke protocol and a follow-up MRI scan post-thrombolysis to demonstrate restoration of perfusion in the ischemic penumbra. On follow-up MRI brain scans, about 86% of cases showed the opening up of thrombosed vessels with the resultant restoration of parenchymal perfusion in the penumbra, and the majority of them had a good clinical outcome.

Six patients (14%) did not undergo thrombolysis due to several reasons, including one patient being on ventilator support, three patients being discharged against medical advice (DAMA), and two patients refusing thrombolysis due to financial constraints (Table 6).

**Table 6:** Showing the management of ischemic stroke in the study group.

Management of ischemic stroke	Number	Percentage
Underwent thrombolysis	37	86%
Did not undergo thrombolysis	6	14%

## Discussion

Ischemic stroke occurs as a result of a reduction in blood flow to a critical level in a specific volume of brain tissue. After this decline occurs, a sequence of biochemical changes begins that, if not reversed, will lead to neuronal death [8]. However, the loss of function due to ischemia can be reversed. The timeframe for recovery of ischemic symptoms is directly related to the level of reduced blood flow. Severe underperfusion with cerebral perfusion values <10 ml/100 g/min can lead to infarction within minutes; however, more moderate levels of ischemia (10-20 ml/100 g/min) may recover for a period of time [9].

Blood flow to the infarct area is very low, leading to rapid cell death, and there is a peripheral zone of penumbra where flow declines more moderately and cell death does not ensue instantly. Penumbra is salvageable tissue that could lead to an infarction. However, if blood flow is normalized at the appropriate time, it can be salvaged [10].

**Figure 1:**
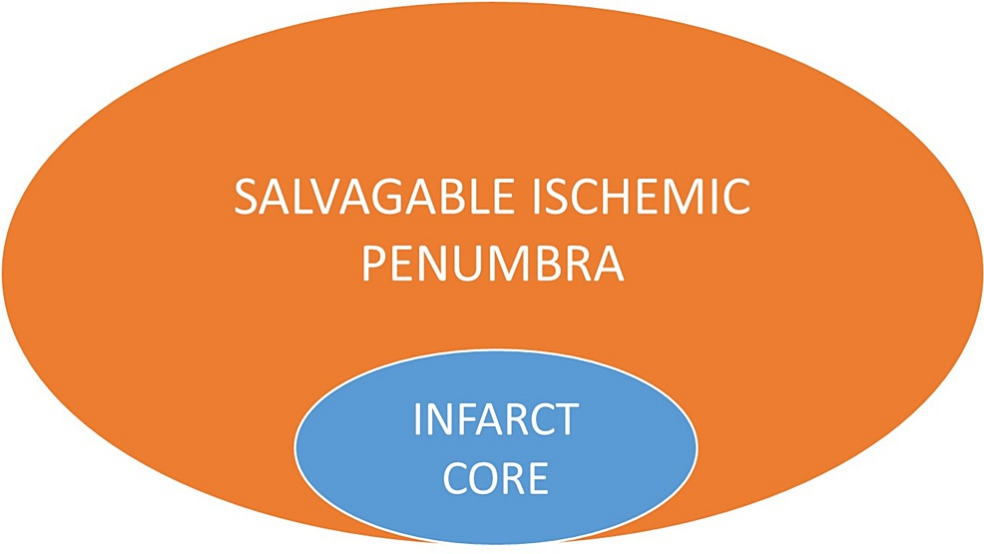
Schematic representation of ischemic penumbra. The inner blue circle represents the infarct core/cell death and the outer orange circle represents salvageable ischemic penumbra (target of thrombolysis) that is marginally perfused and metabolically unstable.

The information regarding the tissue at risk around the infarct core is of the utmost importance in the management and prediction of outcomes. Diffusion-perfusion mismatch indicates a larger perfusion defect than the diffusion-restricted area, which in turn indicates an ischemic penumbra (Figure 1).

Arterial spin labelling measures tissue perfusion (blood flow) using magnetically labelled arterial blood water protons as an endogenous tracer that aids in identifying the penumbra. ASL is well suited for perfusion studies in healthy subjects, patients with renal impairment, and those requiring repeated monitoring. It is also an impressive method to study perfusion in children where the use of exogenous contrast agents may be limited. One advantage of ASL over traditional bolus techniques is that it can be quantified.

Limitations of ASL

Small zones of ischemic penumbra cannot be assessed, and they are prone to movement artifacts. ASL may also overestimate the penumbra in physiological areas of hypoperfusion [11-13]. Previous studies have also stated that ASL can depict large perfusion defects and can predict the clinical outcome of acute ischemic stroke when combined with DWI [14-16] (Table 7).

**Table 7:** Review of literature. ASL: arterial spin labelling, DSC: dynamic susceptibility contrast.

S.No	Author	Year	Findings
1.	Bokkers et al. [14]	2012	ASL can depict large perfusion deficits and perfusion–diffusion mismatches in correspondence with DSC
2.	Bivard et al. [15]	2013	ASL hyperperfusion at 24 hours was associated with better clinical outcomes in acute stroke
3.	Thamm et al.[16]	2019	Adding perfusion imaging to structural imaging and clinical data significantly improved outcome prediction when added to standard stroke protocol

The current study showed that ASL was successful in detecting the ischemic penumbra and helped in the clinical management of acute ischemic stroke. The majority of the cases diagnosed to have a DWI-ASL mismatch who underwent timely thrombolysis had a good clinical outcome. Out of 37 patients who underwent thrombolysis, 15 patients had a modified Rankin Score (mRS) of 0, 10 patients had a score of 1, 8 patients had a score of 2, 2 patients had a score of 3, and 2 patients had a mRS score of 4 (Table 8).

**Table 8:** Clinical outcome of the patients who underwent timely thrombolysis. Modified Rankin Score (mRS) description: 0: no symptoms/normal (physical, cognitive etc.). 1: no significant disability despite symptoms; able to carry out all usual duties and activities. 2: slight disability; unable to carry out all previous activities, but able to look after own affairs without assistance. 3: moderate disability; requiring some help, but able to walk without assistance from another individual (use of walking aids alone is not counted as assistance). 4: moderately severe disability; unable to walk without assistance and unable to attend to own bodily needs without assistance. 5: severe disability; bedridden, incontinent and requiring constant nursing care and attention. 6: dead.

No. of patients	Modified Rankin Score (mRS)
15	0
10	1
8	2
2	3
2	4

**Figure 2:**
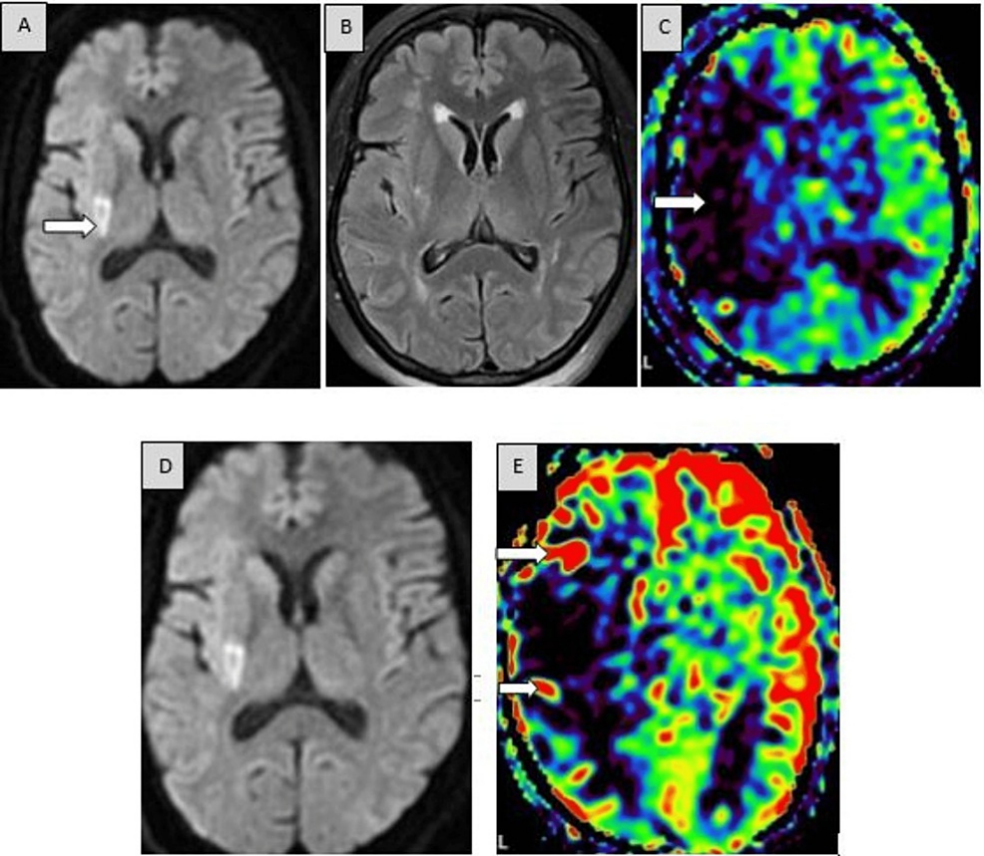
Pre- and post-thrombolysis images showing right MCA infarct with restoration of perfusion on timely thrombolysis. (A) MRI brain DWI shows a small area of diffusion restriction in the right posterior putamen (arrow). (B) Axial FLAIR image; note no significant corresponding signal changes seen in the right putamen, suggestive of a DWI-FLAIR mismatch (represents an infarct within the window period). (C) Axial 3D color coding of ASL demonstrates a large perfusion defect (arrow) in the right cerebral hemisphere in the MCA territory out of proportion to the infarct volume (A), suggestive of a DWI-ASL (perfusion) mismatch, i.e., a large peri-infarct potentially salvageable ischemic penumbra. (D) DWI in the same patient, thrombolyzed in the window period. Post-thrombolytic DWI and ASL images show the volume of infarct in the right putamen remains the same with no further progression. (E) ASL shows restoration of perfusion in the ischemic penumbra zone in the right cerebral hemisphere (arrows).

**Figure 3:**
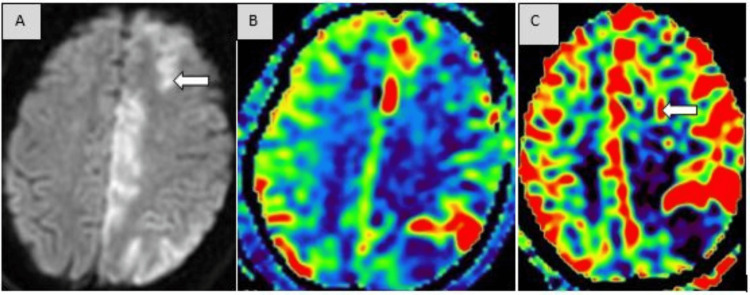
Showing infarct in ACA-MCA watershed infarct with restoration of perfusion on timely thrombolysis. (A) MRI brain axial sections on DWI show an acute ischemic infarct in the left fronto-parietal region (arrow) involving ACA-MCA territory. (B) ASL image showing a large ischemic penumbra in the left fronto-parietal lobe. (C) Effective restoration of perfusion (arrow) after thrombolysis in the previously hypoperfused ischemic penumbra.

The following images demonstrate different cases of acute ischemic infarction pre- and post-thrombolysis involving the MCA territory (Figures 2A-2E) and the ACA-MCA territory (Figures 3A-3C).

Some of the limitations of this study are the small and heterogeneous sample group and selection bias, so as to include only those within the window period with a DWI-FLAIR mismatch. Other limitations are that ASL is predominantly sensitive to grey matter perfusion and difficult to assess white matter lesions. Due to the limited signal-to-noise ratio, it was therefore difficult to detect small cerebral blood flow changes in white matter with ASL. Another limitation is artefacts, which create uncertainty in acute stroke as territorial infarcts might also be expected to present identical findings. The current study is also limited by the unavailability of a high magnetic field strength MR scanner (3 Tesla) and quantification software for ASL parameters.

Other uses of ASL

There are various applications of ASL, as it not only helps in the diagnosis of stroke but can also be utilized in detecting and grading tumours, recurrence of tumours, mesial temporal lobe sclerosis and other causes of seizures, neurodegenerative disorders, migraine, and a few other uses of ASL to name a few [17].

## Conclusions

ASL plays a crucial role in identifying the ischemic penumbra and in the management of ischemic stroke. Timely intervention (i.e., within the window period) using thrombolytic agents prevents the progression of ischemic penumbra into irreversible infarction, thereby providing the patient with a good quality of life.

Although ASL has been around for more than two decades, it only recently began to make the transition from a research tool to clinical use due to the increasing awareness of its capabilities among radiologists and clinicians, which has made this approach more reliable on MR imaging platforms. The experience with ASL in this study population is encouraging and suggests that the time may be ripe to consider including ASL in imaging-based stroke and other pathologies to understand its practical utility.
